# The HIV Protease Inhibitor Ritonavir Reverts the Mesenchymal Phenotype Induced by Inflammatory Cytokines in Normal and Tumor Oral Keratinocytes to an Epithelial One, Increasing the Radiosensitivity of Tumor Oral Keratinocytes

**DOI:** 10.3390/cancers17152519

**Published:** 2025-07-30

**Authors:** Silvia Pomella, Lucrezia D’Archivio, Matteo Cassandri, Francesca Antonella Aiello, Ombretta Melaiu, Francesco Marampon, Rossella Rota, Giovanni Barillari

**Affiliations:** 1 Department of Clinical Sciences and Translational Medicine, University of Rome Tor Vergata, 00133 Rome, Italy; silvia.pomella@uniroma2.it (S.P.);; 2 Department of Hematology and Oncology, Cell and Gene Therapy, Bambino Gesù Children’s Hospital, Istituto di Ricovero e Cura a Carattere Scientifico, 00146 Rome, Italy; 3 Department of Experimental Medicine, Sapienza University of Rome, 00161 Rome, Italy; 4 Department of Radiological Oncological and Pathological Sciences, Sapienza University of Rome, 00161 Rome, Italy; 5 Departmental Faculty of Medicine, Saint Camillus International University of Health and Medical Sciences, 00131 Rome, Italy

**Keywords:** inflammation, interleukins, AKT, EMT, cell survival, cell invasion, OPMD, OSCC, ritonavir, radiosensitivity

## Abstract

**Background/Objectives:** During the repair of a wounded epithelium, keratinocytes become invasive via the epithelial-to-mesenchymal transition (EMT) process. Usually temporary and controlled, EMT persists in a chronically inflamed epithelium and is exacerbated in epithelial dysplasia and dysregulated in invasive carcinomas. Here we investigated the effects that IL-1 beta, IL-6, and IL-8, inflammatory cytokines expressed in specimens from OPMDs and OSCCs, have on NOKs and OSCC cells. Methods: AKT activation and EMT induction were assessed along with cellular invasiveness. **Results:** IL-1 beta, IL-6, and IL-8 induced EMT in NOKs, ex novo conferring them invasive capacity. The same cytokines exacerbated the constitutive EMT and invasiveness of OSCC cells. Since these phenomena were accompanied by AKT activation, we tested whether they could be influenced by RTV, a long-used anti-HIV drug that was previously found to block the activation of human AKT and exert antitumor effects. We observed that therapeutic amounts of RTV counteract all the above-mentioned tumorigenic activities of ILs. Finally, consistent with the key role that AKT and EMT play in OSCC radio-resistance, RTV increased OSCC cells’ sensitivity to therapeutic doses of ionizing radiation. **Conclusions:** These preliminary in vitro findings encourage the use of RTV to prevent the malignant evolution of OPMDs, reduce the risk of OSCC metastasis, and improve the outcomes of anti-OSCC radiotherapy.

## Simple Summary

Consistent with the protumor role of chronic inflammation, interleukin (IL)-1 beta, IL-6, and IL-8 expressed in oral potentially malignant disorders (OPMDs) increase when the latter evolve into oral squamous cells carcinoma (OSCC) and, even more, when OSCC metastasizes. Based on these data, we evaluated ILs’ effect on the phenotype of human normal oral keratinocytes (NOKs) and OSCC tumor cells. We found that IL-1 beta, IL-6, and IL-8 trigger AKT signaling in both NOKs and OSCC cells, ex novo inducing NOKs’ invasiveness and exacerbating OSCC cells’ invasive behavior. In this context, ritonavir (RTV), a HIV protease inhibitor that was previously reported to inhibit human AKT activation, counteracted the pro-invasive effects of ILs and increased the sensitivity of OSCC cells to ionizing radiation. In addition to providing insight into how inflammation contributes to OSCC onset and progression, these preliminary in vitro findings support the use of RTV in the treatment of OSCC, an invasive and radioresistant malignancy.

## 1. Introduction

To heal a wound, epithelial cells must lose their static, apically–basally oriented phenotype and acquire a mobile and invasive one [1,2,3,4]. This occurs through the epithelial-to-mesenchymal transition (EMT), a multistep process that is promoted by a variety of cytokines, including inflammatory ones [5,6,7].

In short, by binding to cell membrane receptors, these cytokines trigger in epithelial cells intracellular signaling pathways, among which the phosphoinositide 3 kinase/protein kinase B (AKT) is particularly relevant to the onset and maintenance of EMT [8,9,10,11]. In fact, upon its phosphorylation, AKT activates transcription factors such as those belonging to the zinc finger E-box-binding homeobox, basic helix–loop–helix twist homolog, and/or zinc finger snail homolog families [3,4,8,12]. These transcription factors, in turn, repress the expression of the intercellular adhesion molecule epithelial cadherin (E-cadherin), thereby loosening the strong adhesiveness that normally exists between epithelial cells [4,8]. At the same time, the abovementioned transcription factors induce in epithelial cells the synthesis of proteins that are typical of mesenchymal cells. Amidst such proteins are Vimentin (VIM), a cytoskeletal component actively involved in cell migration, and the matrix metalloproteinase (MMP)-9, a proteolytic enzyme that efficiently degrades the basement membranes and extracellular matrix: this endows epithelial cells with invasive abilities [3,4,12].

While a transient, reversible, and tightly controlled EMT accompanies physiological tissue remodeling or repair, a dysregulated and persistent EMT is associated with inflammatory, degenerative, or neoplastic diseases [13,14]

Among the latter are the squamous cell carcinomas (SCCs) of the pharynx, larynx, or oral cavity [15,16]. Taken together, the SCCs of the head and neck region represent the sixth most common malignancies worldwide [17]. Among the SCCs of the oral cavity and oropharynx (OSCCs), they account for over 90% of oral cancers [18].

The onset of OSCCs often follows a stepwise progression from hyperplastic/dysplastic lesions that as whole are termed oral potentially malignant disorders (OPMDs), since they exhibit varying risks of malignant transformation [19,20,21,22].

Several studies have emphasized the key role of chronic inflammation in the development of OPMDs, as well as in their progression to OSCCs [12,23,24,25,26].

Specifically, in the oral cavity and oropharynx, inflammation may be triggered by physical, chemical, and/or microbial agents wounding the oral mucosa [20,22,24]. Such damage is followed by local infiltration of activated leukocytes that, together with lining epithelial cells, produce and release inflammatory mediators and growth factors [23,24,27]. Those cytokines, in turn, induce the oral epithelial cells that survived the damage to undergo EMT [24]. If pathogenic stimuli persist, chronic mucositis occurs, which maintains and exacerbates EMT [24]. In such a context, a perduring dedifferentiation of epithelial cells, together with their long-lasting invasiveness, may favor the development or progression of carcinomas [28]. Indeed, downregulated E-cadherin and newly induced Vimentin characterize dysplastic and hyperplastic OPMDs [23,29]. In agreement with these histologic features, the proinflammatory cyclooxygenase/prostaglandin E2 pathway is activated in OPMDs [25,26]. Moreover, the inflammatory cytokines interleukin (IL)-1 beta, IL-6, and IL-8 are overexpressed in OPMDs and found in the saliva of individuals bearing the lesions [30,31,32,33]. Consistent with local inflammation, phosphorylated AKT (pAKT) is expressed in OPMDs, while it is not detectable in healthy oral mucosa [34].

A further progressive increase in IL levels in both saliva and oral mucosa, and the consequent intensification of AKT activation, accompany the evolution of OPMDs into invasive and metastatic OSCCs [2,12,23,24,29,33,34].

Notably, pAKT levels continue to rise in parallel with OSCC clinical progression [34,35]. As a consequence, in OSCCs, the inflammation-induced EMT is exacerbated, and ultimately becomes dysregulated: the loss of E-cadherin and the acquisition of VIM and MMP-9 are hallmarks of OSCCs and are correlated with the clinically aggressive behavior of these tumors, namely their rapid and remarkable growth, invasion, recurrence, and metastasis [1,2,23,36].

Currently, OSCC treatment involves surgical resection, immunotherapy, chemotherapy, radiotherapy, and/or a combination thereof [37,38]. Particularly concerning radiotherapy, it is applied after surgery, or it is the treatment of choice for advanced-stage, surgically unresectable OSCCs [37,39]. However, over time, OSCCs can become radioresistant: this severely reduces the survival rate of OSCC patients, which has not changed over the last sixty years, remaining around 50% [39,40].

Therefore, it is urgent to identify new anti-OSCC therapeutic protocols, possibly more effective than those used today.

In this regard, it must be highlighted that the radioresistance of OSCC cells is associated with their constitutive AKT activation and EMT phenotype, which strengthen the viability of these tumor cells [41,42].

Regarding this, previous work has shown that ritonavir (RTV), a human immunodeficiency virus (HIV) protease inhibitor that has long been successfully used to treat HIV-infected individuals, counteracts the phosphorylation of AKT in human cells [43,44,45,46]. Such an ability of RTV contributes, at least in part, to the unpredicted antitumor effects that this anti-HIV drug has exerted, even in the absence of HIV-1, in a wide variety of preclinical or clinical models of neoplasia, SCC included [43,44,45,46,47,48,49,50,51].

Based on this body of data, here we evaluated whether RTV concentrations as found in the plasma of treated patients could inhibit EMT in normal oral keratinocytes (NOKs) or in tumor cells derived from human OSCCs (OSCC cells), and whether this is associated with a mitigation of the highly invasive phenotype of OSCC cells and/or with their increased sensitivity to ionizing radiation (IR).

## 2. Materials and Methods

### 2.1. Cell Lines

Normal oral keratinocytes (NOKs, human gingiva, FC-0094) and their growth medium with supplements and serum (LL-0007) were purchased from Lifeline Cell Technologies (Frederick, MD, USA). SSC-25 cells (Tongue SCC, CRL-1628) and Detroit 562 cells (Oropharynx SCC, CCL-138) were obtained from the American Type Culture Collection (ATCC, Rockville, MD, USA) and cultured in RPMI 1640 and DMEM high glucose, respectively. Both media were from Invitrogen (Carlsbad, CA, USA), and they were supplemented with 10% fetal bovine serum (FBS), 1% L-glutamine, and 1% penicillin-streptomycin. Cells were cultured at 37 °C in a humidified atmosphere of 5% CO_2_/95% air, and they were regularly checked for mycoplasma contamination.

### 2.2. Reagents

RTV (SML0491) was purchased from Sigma-Aldrich (St Louis, MO, USA) and suspended in DMSO. Human recombinant IL-1 beta (130-093-897), IL-6 (130-093-931), and IL-8 (130-122-359) were obtained from Miltenyi Biotec (Bergisch Gladbach, Germany), reconstituted in deionized sterile-filtered water, and diluted in cold phosphate-buffered saline (PBS)—0.1% bovine serum albumin (BSA). Monoclonal antibodies directed against E-cadherin (cat. n. 3195), Vimentin (cat. n. 5741), AKT (cat. n. 4691), and polyclonal antibodies raised against pAKT Ser473 (cat. n. 9271) and gamma H2AX Ser139 (cat. n. 2577) were obtained from Cell Signaling Technology (Danvers, MA, USA). Anti-Vinculin (hVIN-1; cat. n. V9131) monoclonal antibody was from Sigma-Aldrich (St Louis, MO, USA).

### 2.3. Treatments

NOKs, SCC-25, and Detroit cells were treated every 48 h for 7 days with either a combination of IL-1 beta, IL-6, and IL-8 (referred to as ILs) at 10 ng/mL each, or with the IL dilution buffer (PBS—0.1% BSA) as a control.

For combinatorial treatments, NOKs, SCC-25, and Detroit cells were similarly treated every 48 h for 7 days with ILs (10 ng/mL each) or the corresponding vehicle control. Subsequently, cells were treated daily for 6 days with either 10 μM RTV or its vehicle (DMSO) as a control.

### 2.4. Quantitative Real-Time PCR

Total RNA was extracted using the RNeasy Mini Kit (74104, Sigma-Aldrich, St Louis, MO, USA). The reverse transcription was performed using the Improm-II Reverse Transcription System (A3800, Promega, Madison, WI, USA). TaqMan gene assays (Applied Biosystems, ThermoFisher Scientific, Waltham, MA, USA) for human *E-Cadherin* (*CDH1*, Hs01023895_m1), *Vimentin* (*VIM*, Hs00958111_m1), and *MMP9* (Hs00957562_m1) were used. Human *GAPDH* (Hs99999905_m1) was the reference gene for mRNA level normalization. Applied Byosistems QuantStudio 7 Pro (Applied Biosystems, ThermoFisher Scientific, Waltham, MA, USA) was employed for measurements. Data were calculated by the 2^−ΔΔCt^ method. Experiments were carried out in triplicate.

### 2.5. Western Blotting

Western blotting was performed as previously described [52]. Briefly, whole-cell lysates were obtained by homogenizing cells in RIPA lysis buffer (50 mM Tris pH 7.4, 150 mM NaCl, 1% Triton X-100, 1 mM EDTA, 1% sodium deoxycholate, 0.1% SDS), supplemented with a protease inhibitor cocktail (Sigma-Aldrich, St Louis, MO, USA), NaF 1 mM, Na_3_VO_4_ 1 mM, and PMSF 1 mM. Protein contents were quantified with the BCA Protein Assay Kit (Pierce, ThermoFisher Scientific, Waltham, MA, USA) according to the manufacturer’s protocol and boiled in reducing SDS sample buffer. Total proteins were then separated onto SDS-PAGE and transferred onto filters that were probed with the indicated primary antibodies and, finally, with species-specific secondary antibodies (anti-mouse, cat. n. 7076, or anti-rabbit, cat. n. 7074, Cell Signaling Technology, Danvers, MA, USA). ECL Western Blotting Detection Reagents (Amersham, GE HEALTHCARE BioScience Corporate, Piscataway, NJ, USA) were used for signal detection, and ChemiDoc XRS+ (BIORAD, Hercules, CA, USA) was employed for image acquisition. Experiments were carried out in triplicate.

### 2.6. Immunofluorescence

NOKs, SCC-25 cells, and Detroit cells were fixed with 4% paraformaldehyde/PBS for 15 min at room temperature, washed 3 times with PBS, permeabilized in 0.3% Triton X-100/PBS for 5 min at room temperature, and incubated with the indicated primary antibody, as reported in [53]. Antibody binding was revealed using species-specific secondary antibodies coupled to Alexa Fluor 488 or 555 (Invitrogen, Carlsbad, CA, USA). DAPI (D9542, Sigma-Aldrich, St Louis, MO, USA) was employed to counterstain the nuclei. Images were acquired with a Leica DMi8 microscope (Leica microsystems, Mannhein, Germany). Experiments were carried out in triplicate.

### 2.7. Measurement of MMP-9 Activity

MMP-9 activity on NOK cells was measured using a Human Active MMP-9 Fluorokine E kit (F9M00; R&D Systems, Minneapolis, MN, USA) according to the manufacturer’s instructions. Relative fluorescence units (RFUs) were measured using BioTek Synergy H1 multimode reader (Agilent Technologies, Santa Clara, CA, USA).

### 2.8. Zymography

Media from SCC-25 and Detroit cells were collected, and 40 μL/well were applied to 10% SDS-PAGE gels co-polymerized with 1% gelatin (G2500, Sigma-Aldrich, St Louis, MO, USA). After electrophoresis under non-reducing conditions, the gels were washed three times in 4% Triton X-100, incubated for 24 h at 37 °C in 50 mM Tris, 0.02% NaN_3_, and 10 mM CaCl_2_, and then stained with Coomassie brilliant blue (ThermoFisher Scientific, Waltham, MA, USA). Regions of gelatinolytic activity were observed as clear bands against a blue background. Experiments were carried out in triplicate.

### 2.9. Migration and Invasion Assays

Migration assays were performed employing transwell permeable supports with an 8.0 µm PET membrane (353097, Corning, NY, USA). A total of 1.5 × 10^5^ cells were seeded in the upper portion of the transwell, separated by a PET membrane from the lower compartment, containing DMEM with 10% FBS, which was employed as chemoattractant [54]. After incubation at 37 °C for 18 h (SCC-25 and Detroit cells) and 24 h (NOKs), migrated cells were stained with crystal violet staining solution (0.5%, Sigma-Aldrich, St Louis, MO, USA). The ImageJ 1.52q software quantified the number of migrated cells. For the invasion assays, the same procedure employed for the migration tests was adopted, with the difference that the upper portion of the transwell was coated with 50 μL of 0.3 mg/mL of growth factor-reduced Matrigel (354230, Corning, NY, USA). Experiments were carried out in triplicate.

### 2.10. Cell Viability Assays

SCC-25 and Detroit cells were seeded into T25 flasks at a density of 1.5 × 10^5^ cells/flask. Trypan blue dye exclusion test was used to evaluate cell viability. The Countess II Automated Cell Counter (ThermoFisher Scientific, Waltham, MA, USA) assessed the number of cells. Experiments were carried out in triplicate.

### 2.11. In Vitro Irradiation and Colony Formation Assay

In vitro irradiation was delivered at room temperature using the Radgil2 X-Ray irradiator (Gilardoni, Mandello del Lario, Italy). Colony formation assays were performed as in [55]. Briefly, SCC-25 and Detroit cells were irradiated with a single dose of 4 Gy and counted six hours post irradiation. Then, 1 × 10^3^ cells were plated in growth medium in triplicate in 6-well tissue culture plates. The medium was refreshed once a week, and after two weeks, cells were fixed and stained with crystal violet staining solution (0.5%). Colonies containing >50 cells were counted. Experiments were carried out in triplicate.

### 2.12. Cell Cycle Assay

SCC-25 and Detroit cells were analyzed by flow cytometry as previously reported [56]. In short, cells were harvested by trypsinization, washed in cold PBS, and fixed overnight at 4 °C in a 1:1 solution of PBS and acetone/methanol (1:4 *v*/*v*). Then, cells were pelleted for 5 min at 1200 rpm and were stained with a 1:1 solution of propidium iodide (100 μg/mL; P4170, Sigma-Aldrich, St Louis, MO, USA) and PureLink RNaseA (2 mg/mL; 12091021, Invitrogen, Carlsbad, CA, USA) for 30 min at room temperature in the dark. Stained cells were acquired and analyzed using FACSCanto II equipped with a FACSDiva 6.1 CellQuestTM software (Becton Dickinson Instrument, San Josè, CA, USA). Experiments were carried out in triplicate.

### 2.13. Statistical Analysis

The two-tailed Student’s *t*-test was utilized for comparison between two groups; one-way ANOVA was applied for comparison among more than two groups; and two-way ANOVA was applied to analyze data with multiple variables. Statistical significance was set at *p*-values < 0.05. GraphPad Prism 8.4.3 (Dotmatics, San Diego, CA, USA) was used to perform all the analyses.

## 3. Results

### 3.1. IL-1 Beta, IL-6, and IL-8 Induce Mesenchymal Traits and Abilities in Primary Human NOKs

Initial experiments evaluated the effect that IL-1 beta, IL-6, and IL-8, inflammatory cytokines that are upregulated in OPMDs [30,31,32,33], have on the phenotype of human primary NOKs. To this end, the keratinocytes were exposed for one, two, four, or seven days to human recombinant IL-1 beta, IL-6, and IL-8, which were combined at 10 ng/mL each (IL-NOKs) (Supplementary Figure S1a). NOKs exposed for the same time periods to the IL suspension buffer were employed as controls (CR-NOKs).

**Figure 1:**
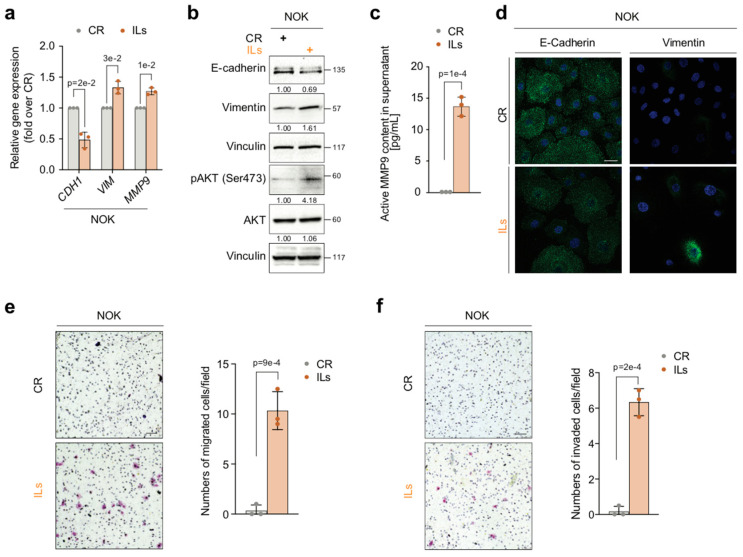
ILs induce mesenchymal traits in NOKs. (**a**) NOKs were treated for 7 days with either IL-1 beta, IL-6, and IL-8 (ILs) combined at 10 ng/mL each or using IL dilution buffer as the control (CR). The mRNA levels of E-cadherin (*CDH1*), Vimentin (*VIM*), and *MMP-9* were assayed by RT-qPCR. Gene expression levels were normalized to *GAPDH* levels and reported as fold increases over the CR (1 arbitrary unit, not reported). Data are presented as mean values  ±  SDs, using Student’s two-tailed *t*-test (n = 3). (**b**) Representative Western blot of the indicated proteins in NOKs treated as in (**a**). Vinculin was the loading control (n = 3). (**c**) Fluorometric activity assay for active MMP9 in supernatants from NOKs treated as in (**a**) (n = 3). (**d**) Representative immunofluorescence of NOKs treated as in (**a**). E-cadherin or Vimentin expression is shown in green, while nuclei were stained in blue with DAPI. Scale bar  =  25 μm. (n = 3) (**e**) (**left**) Representative microscopic images of migration assay of NOKs treated as in (**a**). Scale bar  =  100 μm. (**right**) Histograms depict the number of migrated cells. Data presented as mean values  ±  SDs, using Student’s two-tailed *t*-test. (**f**) (**left**) Representative microscopic images of invasion assay of NOKs treated as in (**a**). Scale bar  =  100 μm. (**right**) Histograms depict the number of invaded cells. Data presented as mean values  ±  SDs, using Student’s two-tailed *t*-test (n = 3). Exact *p*-values are reported in the figure.

Microscopic inspection revealed that 7-day exposure to combined IL-1 beta, IL-6, and IL-8 induced a heterogeneous phenotypic transition of NOK morphology, which changed from polygonal to spindle-shaped, with “long-armed” intercellular connections (Supplementary Figure S1b). As compared to CR-NOKs, IL-NOKs exhibited significant downregulation of the expression of the gene *CDH1* (*p* = 2 × 10^−2^), coding for the epithelial marker E-cadherin, and a concurrent upregulation of the RNA levels of the mesenchymal markers *VIM* (*p* = 3 × 10^−2^) and *MMP-9* (*p* = 1 × 10^−2^) (Figure 1a). Such a change in *CDH1* and *VIM* gene expression, which is typical of EMT [3,4,8], the one that characterizes OPMDs included [2,23,29], was confirmed at the protein level, both as whole lysates (Western blot, Figure 1b) and at the single-cell level (immunofluorescence staining, Figure 1c). Notably, results from assays performed in cell culture supernatants indicated that MMP-9 activity was absent in CR-NOKs, whereas it was induced ex novo by the combined ILs (*p* = 1 × 10^−4^) (Figure 1d). In agreement with the gained mesenchymal traits, IL-NOKs acquired the capability of migrating (*p* = 9 × 10^−4^) (Figure 1e) and invading through a basement membrane (*p* = 2 × 10^−4^) (Figure 1f). In contrast, CR-NOKs displayed none of these abilities (Figure 1e,f), as dictated by their “static” epithelial phenotype.

Consistent with these results, IL-NOKs exhibited the phosphorylation of AKT on Ser473 (Figure 1b), a molecular event that triggers EMT [9,10,11,57].

Altogether, these data indicate that IL-1 beta, IL-6, and IL-8 ex novo induce mesenchymal traits and migratory and invasive abilities in primary human NOKs and that these phenotypic changes are accompanied by the activation (phosphorylation) of AKT.

### 3.2. RTV Counteracts the Mesenchymal Traits Promoted by ILs in NOKs

Based on the RTV ability to turn off human AKT phosphorylation [43,44,45,46], and considering both the importance that AKT activation has in the induction of EMT [10,11] and ILs’ capability of activating AKT in NOKs (Figure 1b), we treated IL-NOKs for 6 days with RTV concentrations (10 μM) corresponding to the peak levels of the drug detected in the plasma of treated patients [46,47,58] (Supplementary Figure S2a). IL-NOKs treated with the RTV vehicle and CR-NOKs were employed as controls.

**Figure 2:**
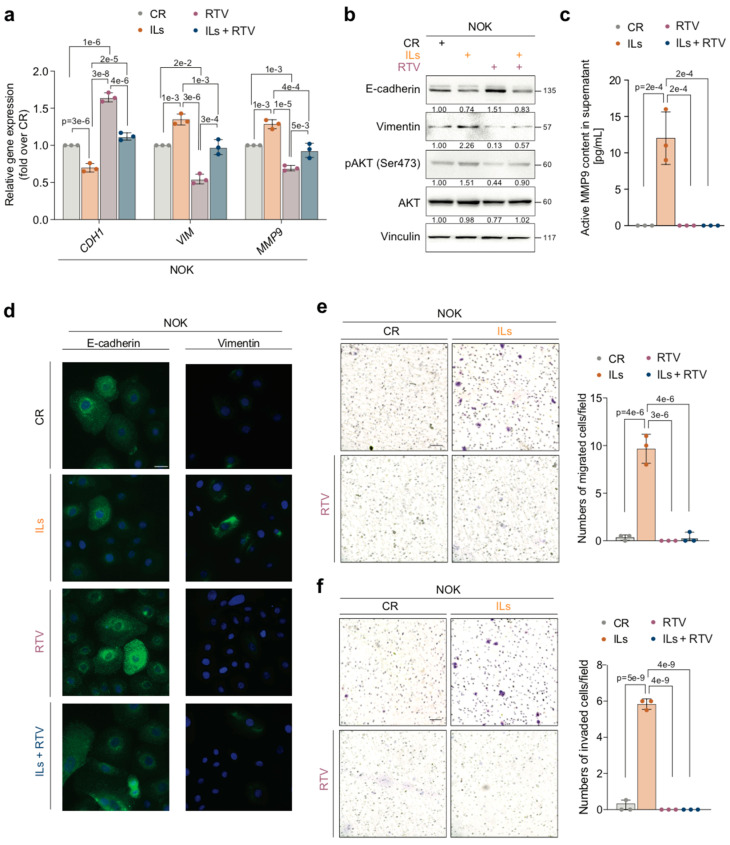
RTV reverses IL-induced mesenchymal traits in NOKs. (**a**) NOKs were treated for 7 days with either IL-1 beta, IL-6, and IL-8 (ILs) combined at 10 ng/mL or with IL dilution buffer each and then exposed for 6 days to 10 μM RTV or its vehicle (DMSO) as a control. The mRNA levels of E-cadherin (*CDH1*), Vimentin (*VIM*), and *MMP-9* were assayed by RT-qPCR. Gene expression levels were normalized to *GAPDH* levels and reported as fold increases over the CR (1 arbitrary unit, not reported). Data are presented as mean values  ±  SDs, using one-way ANOVA (n = 3). (**b**) Representative Western blot of the indicated proteins in NOKs treated as in (**a**). Vinculin was the loading control (n = 3). (**c**) Fluorometric activity assay for active MMP9 in supernatants from NOKs treated as in (**a**) (n = 3). (**d**) Representative immunofluorescence of NOKs treated as in (**a**). E-cadherin or Vimentin expression is shown in green, while nuclei were stained in blue with DAPI. Scale bar  =  25 μm. (n = 3) (**e**) (**left**) Representative microscopic images of migration assay of NOKs treated as in (**a**). Scale bar  =  100 μm. (**right**) Histograms depict the number of migrated cells. Data are presented as mean values  ±  SDs, using one-way ANOVA (n = 3). (**f**) (**left**) Representative microscopic images of invasion assay of NOKs treated as in (**a**). Scale bar  =  100 μm. (**right**) Histograms depict the number of invaded cells. Data are presented as mean values  ±  SDs, using one-way ANOVA (n = 3). Exact *p*-values are reported in the Figure.

Results indicated that treatment with RTV reduced pAKT levels in IL-NOKs (Figure 2b).

Consistent with this finding, RTV converted the mesenchymal-like phenotype of IL-NOKs to epithelial. Specifically, the treatment of IL-NOKs with RTV restored the expression of the *CDH1* gene (*p* = 2 × 10^−5^) while downregulating that of *VIM* (*p* = 1 × 10^−3^) and *MMP-9* (*p* = 4 × 10^−4^), which reached levels as found in CR-NOKs (Figure 2a). Western blot and immunofluorescence staining indicated that RTV also returned the protein expression of E-cadherin and VIM at levels found in CR-NOKs (Figure 2b,c). Moreover, RTV shut off the activity of MMP-9 generated in NOKs by the ILs (Figure 2d). In agreement with these results, treatment with RTV blocked the migratory (*p* = 4 × 10^−6^) and invasive (*p* = 4 × 10^−9^) abilities that combined ILs had induced ex novo in NOKs (Figure 2e,f).

Of interest, RTV also strengthened the epithelial phenotype of CR-NOKs (Figure 2a,b).

### 3.3. IL-1 Beta, IL-6, and IL-8 Exacerbate the Pro-Invasive EMT Phenotype of OSCC Cells

Previous studies reported that the clinical progression of OSCC is accompanied by the exacerbation of the mesenchymal traits of OSCC cells, and by an increase in IL-1 beta, IL-6, and IL-8 tissue and/or salivary levels [12,23,24,33]. To evaluate whether ILs could impact OSCC cell phenotype, we exposed SCC-25 cells (a human cell line derived from a tongue SCC) and Detroit cells (a human cell line derived from an oropharyngeal SCC) for 7 days to combined IL-1 beta, IL-6, and IL-8 (IL-SCC-25 cells and IL-Detroit cells), as performed on NOKs (Supplementary Figure S3a). Cells exposed to the IL suspension buffer were employed as controls (CR-SCC-25 cells and CR-Detroit cells), again as performed on NOKs (Supplementary Figure S3a).

**Figure 3:**
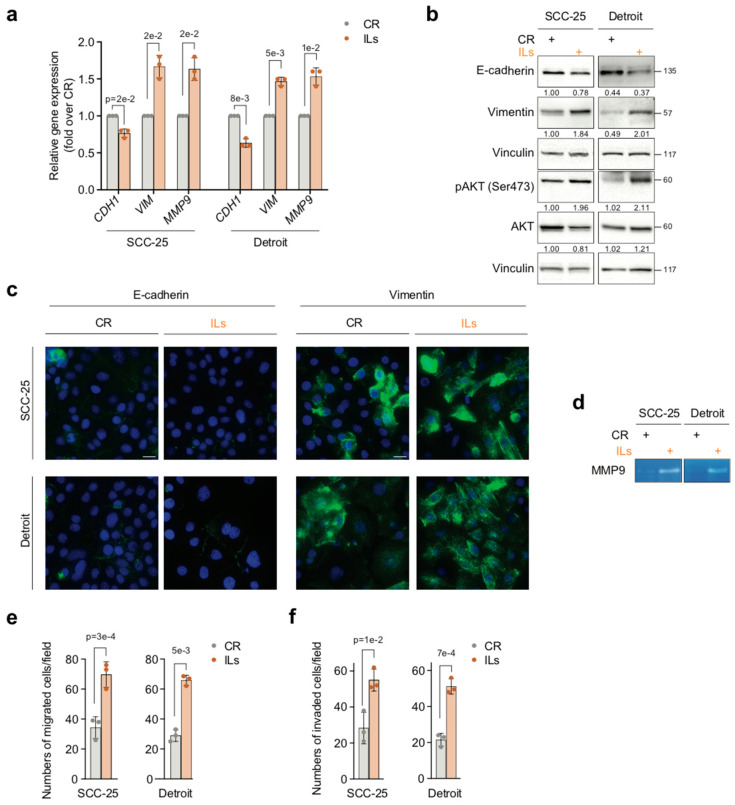
ILs enhance EMT-associated invasiveness in OSCC cells. (**a**) SCC-25 and Detroit cells were treated for 7 days with either IL-1 beta, IL-6, and IL-8 (ILs) combined at 10 ng/mL each or with IL dilution buffer as the control (CR). The mRNA levels of E-cadherin (*CDH1*), Vimentin (*VIM*), and *MMP-9* were assayed by RT-qPCR. Gene expression levels were normalized to *GAPDH* levels and reported as fold increases over the CR (1 arbitrary unit, not reported). Data are presented as mean values  ±  SDs, using the Student’s two-tailed *t*-test (n = 3). (**b**) Representative Western blot of the indicated proteins in SCC-25 and Detroit cells treated as in (**a**). Vinculin was the loading control (n = 3). (**c**) Representative immunofluorescence of SCC-25 and Detroit cells treated as in (**a**). E-cadherin or Vimentin expression is shown in green, while nuclei were stained in blue with DAPI. Scale bar  =  25 μm. (n = 3) (**d**) Representative gelatin zymography of supernatant of SCC-25 and Detroit cells treated as in (**a**) (n = 3). (**e**) Histograms depict the number of migrated SCC-25 cells and Detroit cells treated as in (**a**). Data are presented as mean values  ±  SDs, using the Student’s two-tailed *t*-test (n = 3). (**f**), Histograms depict the number of invaded SCC-25 cells and Detroit cells treated as in (**a**). Data are presented as mean values  ±  SDs, using the Student’s two-tailed *t*-test (n = 3). Exact *p*-values are reported in the Figure.

Although it did not change the morphology of SCC-25 or Detroit cells (Supplementary Figure S3b), 7-day exposure of these cell lines to combined ILs downregulated the expression of the *CDH1* gene (*p* = 2 × 10^−2^ SCC-25 and *p* = 8 × 10^−3^ Detroit) and upregulated that of the *VIM* (*p* = 2 × 10^−2^ SCC-25 and *p* = 5 × 10^−3^ Detroit) and *MMP-9* (*p* = 2 × 10^−2^ SCC-25 and *p* = 1 × 10^−2^ Detroit) genes, as compared to CR-SCC-25 cells or CR-Detroit cells (Figure 3a). In agreement with the mRNA data, Western blot analysis and immunofluorescence staining of IL-SCC-25 cells or IL-Detroit cells showed E-cadherin protein levels to be downregulated, and VIM protein levels to be increased, as compared to the untreated counterparts (Figure 3b,c). Moreover, the combined ILs potentiated MMP-9 proteolytic activity in both SCC-25 and Detroit cells (Figure 3d). As observed in NOKs, these effects were accompanied by the activation of AKT (Figure 3b).

Consistent with the upregulation of VIM expression, a two-fold increase in cellular migration was observed in IL-SCC-25 cells (*p* = 3 × 10^−4^) or IL-Detroit cells (*p* = 5 × 10^−3^), as compared to CR-SCC-25 cells and CR-Detroit cells (Figure 3e). Similarly, in agreement with the enhancement of MMP-9 expression and proteolytic activity, cellular invasion was increased by 2- and 2.5-fold in IL-SCC-25 cells (*p* = 1 × 10^−2^) and IL-Detroit cells (*p* = 7 × 10^−4^), respectively (Figure 3f). In contrast, the exposure of SCC-25 or Detroit cells to ILs had no effect on either cell proliferation or cell cycle distribution (Supplementary Figure S3c,d).

Overall, these results indicate that ILs intensify the mesenchymal, invasive phenotype of OSCC cells, thus providing a possible explanation for the accelerated clinical progression of IL-overexpressing OSCCs [12,23,24,33].

### 3.4. RTV Inhibits the Migration, Invasion, and Growth of OSCC Cells While Countering Their Constitutive or IL-Exacerbated Mesenchymal Phenotype

Considering the RTV capability of turning off AKT phosphorylation [43,44,45,46], we evaluated whether this drug could inhibit EMT in OSCC cells, as found in IL-NOKs. To this end, IL-SCC-25 cells and IL-Detroit cells were cultured for 6 days with 10 μM RTV and then assayed for the RNA expression of E-cadherin (*CDH1*), *VIM*, and *MMP-9*. Cells exposed to the RTV vehicle were employed as controls, as performed on NOKs (Supplementary Figure S4a).

**Figure 4:**
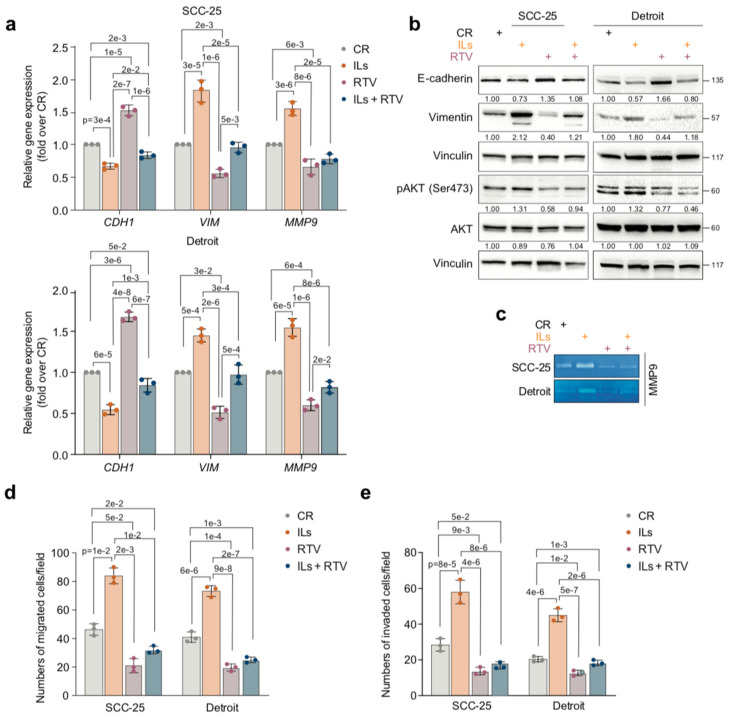
RTV inhibits constitutive tumorigenic and mesenchymal properties and counteracts IL-exacerbated ones in OSCC cells. (**a**) SCC-25 cells and Detroit cells were treated for 7 days with either IL-1 beta, IL-6, and IL-8 (ILs) combined at 10 ng/mL each or with IL dilution buffer and then exposed for 6 days to either 10 μM RTV or its vehicle (DMSO) as a control. The mRNA levels of E-cadherin (*CDH1*), Vimentin (*VIM*), and *MMP-9* were assayed by RT-qPCR. Gene expression levels were normalized to *GAPDH* levels and reported as fold increases over the control (1 arbitrary unit, not reported). Data are presented as mean values  ±  SDs, using one-way ANOVA (n = 3). (**b**) Representative Western blot of the indicated proteins in SCC-25 and Detroit cells treated as in (**a**). Vinculin was the loading control (n = 3). (**c**) Representative gelatin zymography of supernatant of SCC-25 and Detroit cells treated as in (**a**) (n = 3). (**d**) Histograms depict the number of migrated SCC-25 cells and Detroit cells treated as in (**a**). Data are presented as mean values  ±  SDs, using one-way ANOVA (n = 3). (**e**) Histograms depict the number of invaded SCC-25 cells and Detroit cells treated as in (**a**). Data are presented as mean values  ±  SDs, using one-way ANOVA (n = 3). Exact *p*-values are reported in the Figure.

Results indicated that a 6-day culture in the presence of RTV increased E-cadherin (*CDH1*) RNA levels (*p* = 2 × 10^−2^ SCC-25 and *p* = 1 × 10^−3^ Detroit) and reduced those of *VIM* (*p* = 2 × 10^−5^ SCC-25 and *p* = 3 × 10^−4^ Detroit) in IL-SCC-25 cells and IL-Detroit cells, as compared to the same cells cultured in the absence of RTV (Figure 4a). The protein levels of E-cadherin and VIM recapitulated the gene expression data (Figure 4b and Supplementary Figure S4d). In addition, RTV diminished the gene expression and the proteolytic activity of the ECM-degrading, pro-invasive MMP-9 (*p* = 2 × 10^−5^ SCC-25 and *p* = 8 × 10^−6^ Detroit) (Figure 4a,c) in IL-SCC-25 cells or IL-Detroit cells. Consistently, RTV reduced the strong migratory (*p* = 1 × 10^−2^ SCC-25 and *p* = 2 × 10^−7^ Detroit) and invasive (*p* = 8 × 10^−6^ SCC-25 and *p* = 2 × 10^−6^ Detroit) abilities of IL-SCC-25 cells and IL-Detroit cells (Figure 4d,e).

Results from additional work indicated that RTV also reverted the constitutive (innate) mesenchymal, mobile, and invasive phenotype of OSCC cells to a more “epithelial” phenotype. Specifically, analogously to what was observed for IL-treated OSCC cells, 6-day exposure of CR-SCC-25 cells and CR-Detroit cells to 10 μM RTV (compared to the RTV vehicle) increased the RNA and protein levels of E-cadherin (*p* = 1 × 10^−5^ SCC-25 and *p* = 3 × 10^−6^ Detroit), decreased VIM gene and protein expression (*p* = 2 × 10^−3^ SCC-25 and *p* = 3 × 10^−2^ Detroit), and reduced the RNA levels and proteolytic activity of MMP-9 (*p* = 6 × 10^−3^ SCC-25 and *p* = 6 × 10^−4^ Detroit) (Figure 4a–c). Consistently, 6-day exposure to 10 μM RTV (compared to its vehicle) strongly reduced the constitutive migratory (*p* = 5 × 10^−2^ SCC-25 and *p* = 1 × 10^−4^ Detroit) and invasive (*p* = 9 × 10^−3^ SCC-25 and *p* = 1 × 10^−2^ Detroit) abilities of CR-SCC-25 cells and CR-Detroit cells (Figure 4d,e).

Moreover, RTV inhibited the proliferation of SCC-25 and Detroit cells, whether they were IL-treated or not (Supplementary Figure S4b). In accordance, RTV increased the percentage of G1-phase cells, while it decreased the percentage of S- and G2-phase cells, in both IL-treated and untreated SCC-25 and Detroit cells (Supplementary Figure S4c).

All these effects of RTV were accompanied by a strong reduction in pAKT levels in both the cell lines, whether they were IL-treated or not (Figure 4b).

Collectively these data indicate that therapeutic amounts of RTV impair the innate proliferative, migratory, and invasive abilities of OSCC cells and counter the exacerbation of their mesenchymal phenotype promoted by ILs.

### 3.5. Therapeutic Amounts of RTV Sensitize OSCC Cells to IR Therapeutic Doses

Results from previous work indicate that AKT phosphorylation in Ser 473 increases the viability of head and neck SCC cells and hampers IR ability to cause the death of those malignant cells [59]. Since RTV inhibited AKT phosphorylation in SCC-25 and Detroit cells, we evaluated whether the drug could increase the radiosensitivity of these tumor cell lines. To this end, both CR-SCC-25 cells and CR-Detroit cells were cultured for 6 days with 10 μM RTV and then exposed to therapeutic doses (4 Gy) of IR. Cells cultured with the RTV vehicle were used as controls.

Given that IR promotes cell death by damaging the DNA, the phosphorylation of H2AX on Ser139 (gamma H2AX), a well-known sign of DNA damage [60], was assessed in CR-SCC-25 cells or CR-Detroit cells 6 h post irradiation [61].

**Figure 5:**
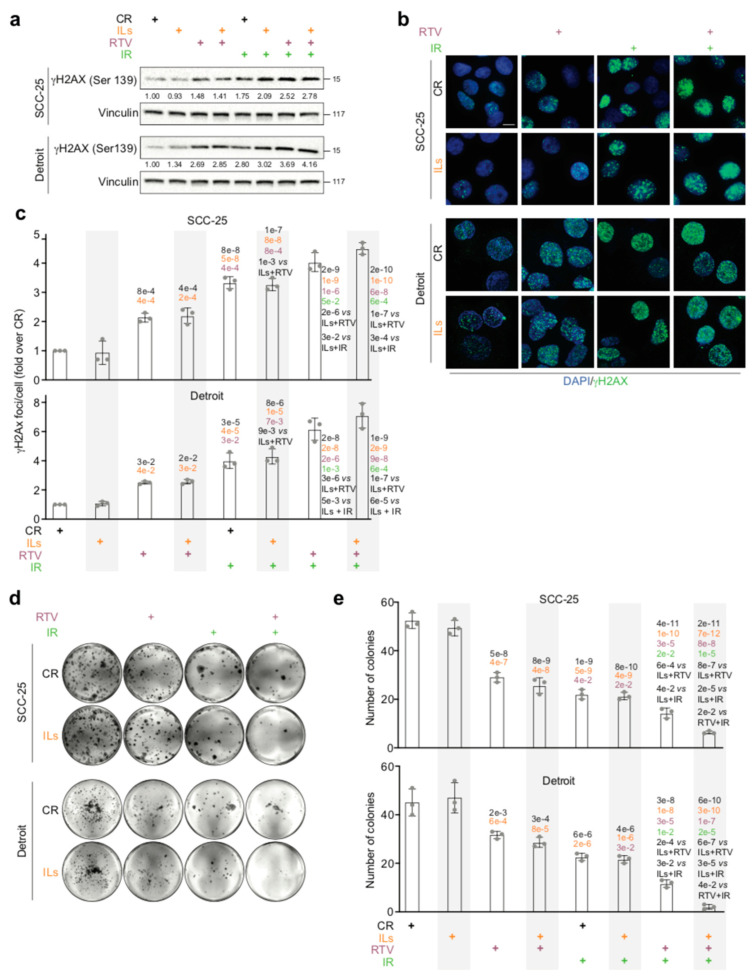
RTV sensitizes OSCC cells to IR. (**a**) SCC-25 cells and Detroit cells were treated for 7 days with either IL-1 beta, IL-6, and IL-8 (ILs) combined at 10 ng/mL each or with IL dilution buffer and then exposed for 6 days to either 10 μM RTV or its vehicle (DMSO) as the control. Cells were then irradiated (4 Gy) and processed 6 h later. Representative Western blot of gamma of the indicated proteins in SCC-25 and Detroit cells treated as in (**a**). Vinculin was the loading control (n = 3). (**b**) Representative immunofluorescence of SCC-25 cells and Detroit cells treated as in (**a**), showing gamma H2AX (Ser139) (green). Nuclei were stained with DAPI (blue). Scale bar  =  10 μm. (**c**) Histograms depict the number of gamma H2AX (Ser139) foci per cell in SCC-25 (**top**) and Detroit (**bottom**) cells treated as in (**a**). Results were expressed as fold increases over control values. Data are presented as mean values  ±  SDs, using one-way ANOVA (n = 3). Black *p*-values (treatment vs. control); yellow *p*-values (treatment vs. ILs); purple *p*-values (treatment vs. RTV); green *p*-values (treatment vs. IR). (**d**) Representative images of colony formation assay of SCC-25 (**top**) and Detroit (**bottom**). (**e**) Histograms depict the number of colonies of SCC-25 (**top**) and Detroit (**bottom**) cells treated as in (**a**). Data are presented as mean values  ±  SDs, using one-way ANOVA (n = 3). Black *p*-values (treatment vs. control); yellow *p*-values (treatment vs. ILs); purple *p*-values (treatment vs. RTV); green *p*-values (treatment vs. IR).

As expected, Western blot analyses indicated that IR increased gamma H2AX levels in both CR-SCC-25 cells and CR-Detroit cells (Figure 5a). Quite surprisingly, however, RTV by itself augmented the levels of gamma H2AX in either of the tested cell lines (Figure 5a). Anyway, gamma H2AX levels increased even more when cells were pretreated with RTV and then exposed to IR (Figure 5a).

Immunofluorescence staining confirmed Western blot data revealing that although both IR and RTV per se significantly augmented the number of gamma H2AX foci, the latter increased to a significantly greater extent when cells were first treated with RTV and then exposed to IR (Figure 5b,c).

Another sign of the decreased viability of an irradiated cell is its reduced clonogenic ability, that is, to duplicate and give rise to colonies of daughter cells [62].

A clonogenic assay was then performed 6 h post IR. Consistent with the data on gamma H2AX, both IR (*p* = 1 × 10^−9^ SCC-25 and *p* = 6 × 10^−6^ Detroit) and RTV (*p* = 5 × 10^−8^ SCC-25 and *p* = 2 × 10^−3^ Detroit) reduced the clonogenic ability of CR-SCC-25 and CR-Detroit cells (Figure 5d,e). Nevertheless, again in agreement with the data on gamma H2AX, such a reduction was much more evident when CR-SCC-25 and CR-Detroit cells were precultured with RTV and then irradiated (*p* = 4 × 10^−11^ SCC-25 and *p* = 3 × 10^−8^ Detroit) (Figure 5d,e).

Additional experiments employed IL-SCC-25 cells and IL-Detroit cells that were irradiated after being cultured with RTV or its vehicle. The results were the same as those obtained with CR-SCC-25 cells or CR-Detroit cells (*p* = 2 × 10^−11^ SCC-25 and *p* = 6 × 10^−10^ Detroit) (Figure 5a–e).

Taken together, these findings indicate that therapeutic doses of RTV increase the sensitivity of OSCC cells to IR.

In this context, it is interesting that although they exacerbate the EMT of SCC-25 cells and Detroit cells, ILs did not increase the resistance of these OSCC cells to the IR lethal effect (Figure 5a–e).

## 4. Discussion

Chronic inflammation is increasingly recognized as a driver of carcinogenesis, mainly because of its capability of promoting genomic instability and fostering a pro-tumorigenic microenvironment [63].

What occurs in the oral cavity and oropharynx reinforces the importance of inflammation in carcinogenesis. In fact, IL-1 beta, IL-6, and IL-8 are overexpressed in OPMD tissues and well detectable in the saliva of people with these premalignant lesions compared to individuals with healthy oral mucosa [30,31,32,33]. This situation becomes even more evident when dysplastic OPMDs evolve into invasive OSCCs [1,2,23,30,31,32,36].

Indeed, IL-1 beta, IL-6, and IL-8 exert activities that could plausibly trigger the onset and/or progression of OSCC. Specifically, IL-1 beta, IL-6, and IL-8 can induce epithelial dysplasia [64,65,66,67]. Moreover, IL-1 beta promotes tumor cell proliferation, survival, and migration [68,69], while IL-6 facilitates tumor immune evasion [70]. On its part, IL-8 enhances tumor cell invasiveness and stimulates the formation of new blood vessels nourishing the growing tumor [23].

Here we have shown that prolonged exposure to combined IL-1 beta, IL-6, and IL-8 deeply affects primary NOKs, in that it changes their morphology from “cobblestone-like” to “spindle-shaped”, reduces E-cadherin levels, and concomitantly induces the synthesis of VIM as well as the expression and proteolytic activity of MMP-9. Notably, these molecular changes are paralleled by the phosphorylation of AKT and correlate with ex novo-induced invasiveness of NOKs, thus further supporting a role for inflammation in OPMDs’ evolution into invasive OSCCs.

These in vitro findings are representative of what is observed in OPMDs and OSCCs and, taken together, suggest that targeting the mechanisms underlying the link between inflammation-driven EMT and OSCC development would offer novel therapeutic strategies against this neoplasm.

In this context, given the key role that AKT signaling plays in both EMT [8,9,10,11,34,35], and the initiation and malignant evolution of OPMDs [34,35], the ability of RTV to counteract the activation of human AKT [43,44,45,46] could make this anti-HIV drug a promising candidate for anti-OSCC therapies.

In support of this hypothesis, we found that therapeutic amounts of RTV impair IL-triggered AKT phosphorylation in NOK cells: this is followed by the restoration of E-cadherin expression and a reduction in VIM levels as well as MMP-9 expression and activity in IL-treated NOKs. Again, consistent with its anti-EMT property, RTV suppresses the migratory and invasive capacities that ILs induced ex novo in NOKs. Altogether, these findings encourage the repurposing of RTV to prevent the onset of OPMDs and, mostly, their evolution into invasive OSCCs.

Still in this framework, one should also consider that OSCC cells constitutively have an EMT-like, mesenchymal phenotype, which makes them very invasive and metastatic [71]. It has been previously reported that IL-1 beta, IL-6, and IL-8 levels in specimens from OSCC patients directly correlate with the clinical aggressiveness of the tumor [12,23,24,33]. Of note, here we have shown that combined IL-1 beta, IL-6, and IL-8 exacerbate the constitutive EMT of OSCC cells, further augmenting the invasive behavior of these malignant cells.

Like NOKs, therapeutic doses of RTV convert the EMT-like, mesenchymal traits of OSCC cells to a more “epithelial” and less invasive phenotype. Such change occurs for both the constitutive and IL-exacerbated EMT of OSCC cells. These results encourage the testing of RTV as a tool to slow the clinical progression of established OSCC.

Again, similar to what has been seen for NOKs, the anti-EMT activity of RTV is also accompanied by the inhibition of AKT phosphorylation in OSCC cells. Concerning this effect of RTV, one should consider that the activation of AKT strengthens the viability of carcinoma cells, hence rendering them more resistant to IR lethal effects [9,59]. Consistent with its inhibitory effect on AKT phosphorylation and in addition to its anti-EMT property, RTV also makes OSCC cells more sensitive to IR. In irradiated OSCC cells, radiosensitivity has here been evidenced by a reduced clonogenic survival fraction and by the accumulation of gamma H2AX, a marker of DNA damage [61]. While the latter finding suggests that RTV is likely to radiosensitize OSCC cells by impairing DNA damage repair mechanisms [61], the radiosensitizing effect of RTV could also depend on its ability to reduce the expression and proteolytic activity of MMP-9. In fact, previous results have indicated that MMP-9 is implicated in tumor radio-resistance as well as tumor metastasis [72].

Interestingly, RTV radiosensitizes OSCC cells regardless of whether they were exposed to IL. This result not only highlights the broad applicability of RTV in OSCC therapy but also suggests that RTV could counteract the radioresistance mediated by the AKT-EMT axis eventually stimulated in an inflammatory tumor microenvironment [73].

These findings, which foster the use of RTV as a radiosensitizing agent for OSCCs, are of clinical importance, considering that over time many OSCCs become radioresistant [39,40].

Although our findings suggest that RTV-mediated inhibition of AKT may contribute to reduced invasiveness and enhanced radiosensitivity, these interpretations remain speculative. Our previous results indicate that in cervical keratinocytes, the inhibition of AKT phosphorylation promoted by RTV reduces the activity of the transcription factor Fra-1, thereby leading to the downregulation of MMP-9 expression [46]. Future investigations will evaluate whether RTV promotes the same phenomenon in NOKs and OSCC cells. While the accumulation of gamma H2AX foci following RTV treatment suggests increased DNA damage or impaired repair, we did not directly investigate the involvement of specific DNA repair pathways such as homologous recombination or non-homologous end joining. Nevertheless, several potential mechanisms could underlie this effect. For instance, RTV-mediated inhibition of AKT signaling may impair non-homologous end joining by affecting the activation of DNA-PKcs, a key enzyme involved in repair of DNA double-strand breaks [74]. Additionally, RTV has been shown to increase oxidative stress, which may overwhelm DNA repair systems and exacerbate DNA damage [75]. Finally, possible proteasome inhibition by RTV could interfere with the turnover and proper regulation of DNA repair proteins [76]. Future studies will be essential to clarify the mechanistic basis of RTV’s effects on DNA damage response and repair.

Definitely, our study has a major limitation, which is that it was conducted entirely in vitro. Therefore, the clinical applications of the results shown here remain theoretical for now. Nonetheless, our findings are corroborated by those of a previous in vivo study reporting that supratherapeutic concentrations of RTV can reduce the growth of and, at the same time, radiosensitize a human laryngeal SCC xenograft [77]. That study also showed that RTV inhibits the proliferation of laryngeal SCC cells in vitro [77]. Here, we have confirmed such findings in OSCC cells. The novelty of the present study mainly consists of having revealed that RTV can counter inflammation-driven steps of oral carcinogenesis such as AKT phosphorylation, EMT and invasiveness of both normal keratinocytes and OSCC cells. In this context, we have found that therapeutic amounts of RTV also block AKT phosphorylation in FaDu cells, a HNSCC cell line that was employed in the above-mentioned in vivo study. This preliminary result, together with our previous finding that RTV counters AKT activation and cellular invasiveness in keratinocytes from uterine SCC [46,47], further encourages RTV exploitation against OSCC. Indeed, histological similarities exist between SCC of the uterine cervix and OPMDs or OSCCs [78,79,80]. Given that OSCCs are very invasive and metastatic [1,2,23,36] and that radiotherapy remains a cornerstone of their management [37,39], the ability of RTV to enhance radiation-induced cytotoxicity while concomitantly suppressing EMT and invasiveness represents a promising therapeutic strategy for this malignancy.

In conclusion, here we have highlighted RTV as an effective anti-mesenchymal, anti-invasive, and radiosensitizing agent for OSCC cells. In this regard, since AKT phosphorylation and EMT diminish the response of carcinoma cells not only to IR but also to cytotoxic drugs [81], our data suggest that RTV could also enhance the efficacy of anti-OSCC chemotherapy. If this were the case, the addition of RTV to cytotoxic drugs and/or IR could augment the survival rate of OSCC patients.

Future studies on these hypotheses are especially recommended if one considers that RTV has been in use for many years and that its pharmacokinetics in humans are well known [58,81,82]: definitely, this will permit a rapid reuse of the drug in patients with OSCC. Furthermore, the RTV patent has expired, and this would have a positive impact on low-income countries and populations.

Finally, it should be emphasized that the easy accessibility of the oral cavity and oropharynx may allow the use of topical preparations of RTV or its derivatives, with a likely reduction in the drug side effects.

## 5. Conclusions

Here we have shown that the proinflammatory cytokines IL-1 beta, IL-6, and IL-8 activate AKT signaling in both NOKs and OSCC cells, ex novo conferring a pro-invasive, mesenchymal-like phenotype to NOKs and exacerbating the constitutive invasiveness of OSCC cells. Of utmost interest, treatment of NOKs or OSCC cells with therapeutic amounts of RTV inhibits IL-promoted AKT phosphorylation, reverts the mesenchymal phenotype to a more “epithelial” one, and blocks MMP-9 activity, hence reducing cellular migration and invasion. Noteworthy, RTV also enhances OSCC cells’ sensitivity to therapeutic doses of ionizing radiation.

Altogether, these results encourage RTV repurposing for countering OSCC development and/or its clinical progression. The long-time established clinical use of RTV, its well-known pharmacokinetics, and the expired patent make this anti-HIV drug particularly attractive for rapid clinical translation. In addition, the ready accessibility of the oral cavity further opens possibilities for topical RTV formulations, potentially minimizing systemic side effects of the drug. Ultimately, combining RTV with standard chemo-radiotherapy could offer a novel, cost-effective strategy to improve therapy outcomes and overall survival in OSCC patients.

## Data Availability

The original contributions presented in this study are included in the article/Supplementary Material.

## Supplementary Materials

The following supporting information can be downloaded at https://www.mdpi.com/article/10.3390/cancers17152519/s1: Figure S1. Effects of ILs on NOKs; Figure S2. Scheme of the treatments on NOKs; Figure S3. Effects of ILs on OSCCs; Figure S4. Scheme of the treatments and effects of RTV on OSCCs.
