# Supplementary material for: The HIV Protease Inhibitor Ritonavir Reverts the Mesenchymal Phenotype Induced by Inflammatory Cytokines in Normal and Tumor Oral Keratinocytes to an Epithelial One, Increasing the Radiosensitivity of Tumor Oral Keratinocytes
Source: Cancers (Basel). 2025 Jul 30;17(15):2519. doi: 10.3390/cancers17152519 (PMC12346649; doi:10.3390/cancers17152519)
Supplement: Supplementary file 1 [file cancers-17-02519-s001.zip › cancers-3748429-supplementary.pdf]

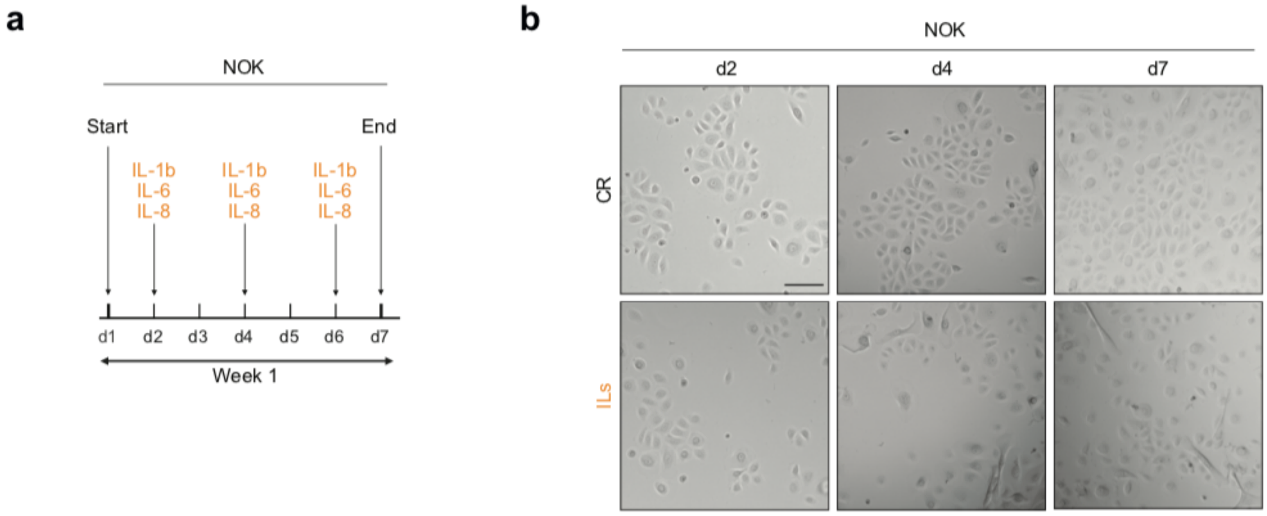

Supplementary Figure S1. Effects of ILs on NOKs.
(a) Schematic model of NOKs treatment with human recombinant IL-1 beta, IL-6 and IL-8, combined at 10ng/mL each. For all time points, NOKs exposed to ILs dilution buffer (PBS-0.1% BSA) were employed as controls (CR). **(b)** Representative images of NOKs treated as in **(a)** at the reported time points. Scale Bar = 100 μm.

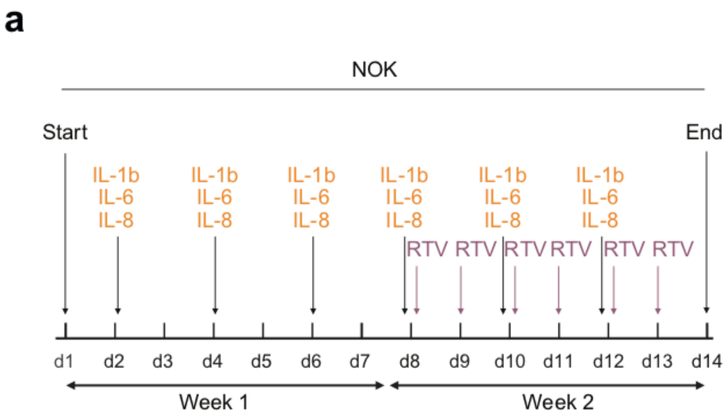

Supplementary Figure S2. Scheme of the treatments on NOKs.
Schematic model of NOKs treatment with combined IL-1 beta, IL-6 and IL-8 (10ng/mL each) and/or 10 μM RTV. NOKs exposed to ILs dilution buffer (PBS-0.1% BSA) and/or RTV vehicle (DMSO) were employed as controls.

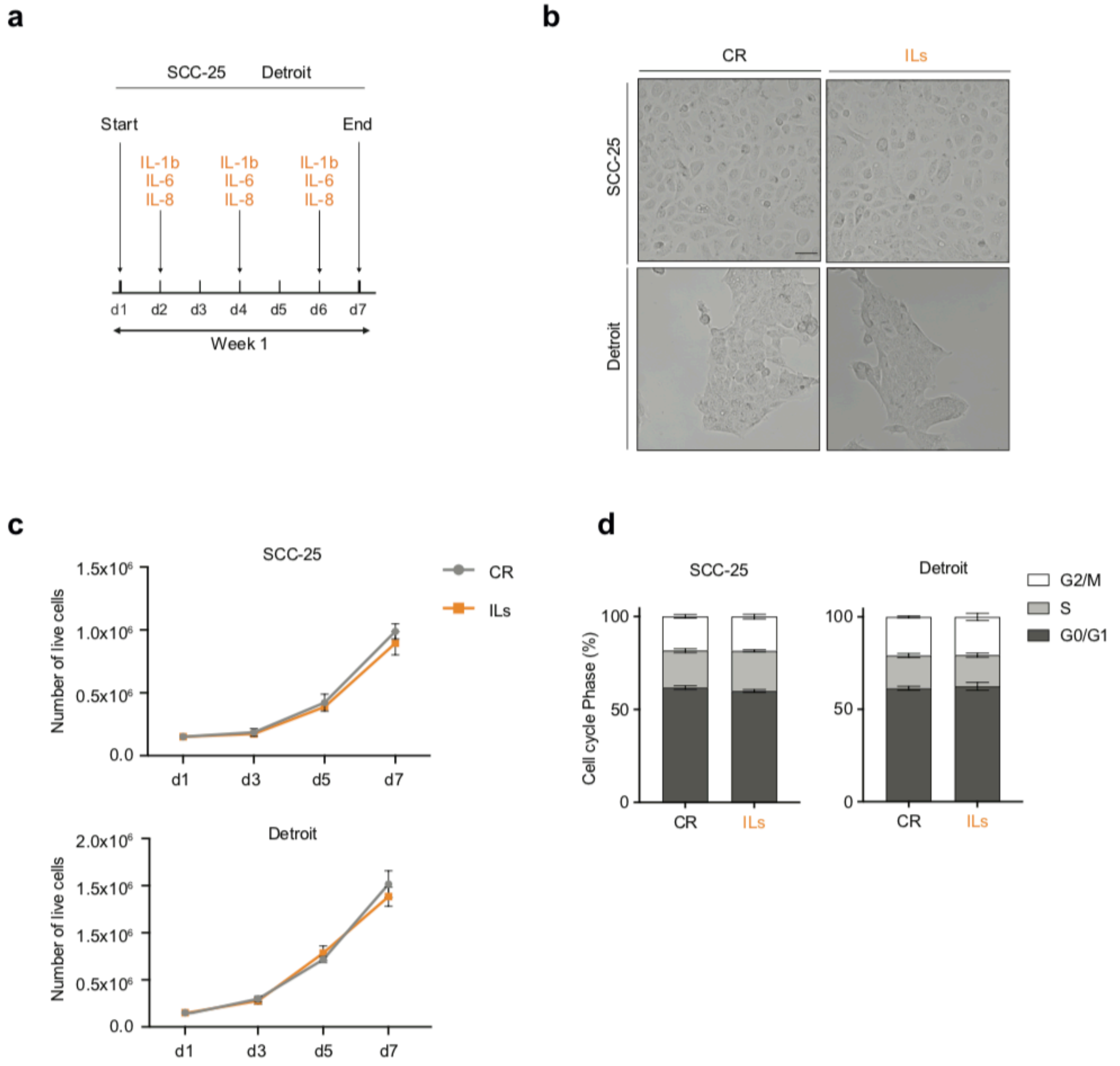

Supplementary Figure S3. Effects of ILs on OSCCs

(a) Schematic model of the treatment of SCC-25 or Detroit cells with combined IL-1 beta, IL-6 and IL-8 (10ng/mL each). Cells exposed to ILs dilution buffer (PBS-0.1% BSA) were employed as controls (CR). **(b)** Representative images of SCC-25 and Detroit cells treated for 7 days as in **(a)**. Scale Bar = 50 μm. **(c)** Growth curve analysis of SCC-25 and Detroit cells treated as in **(a)**. Data are presented as mean values ± SD, two-way ANOVA. **(d)** Histograms depict the percentage of SCC-25 or Detroit cells in G1, S and G2 cell cycle phases on day 7 of the treatment specified in **(a)**. Data presented as mean values ± SD, Student's two-tailed t-test.

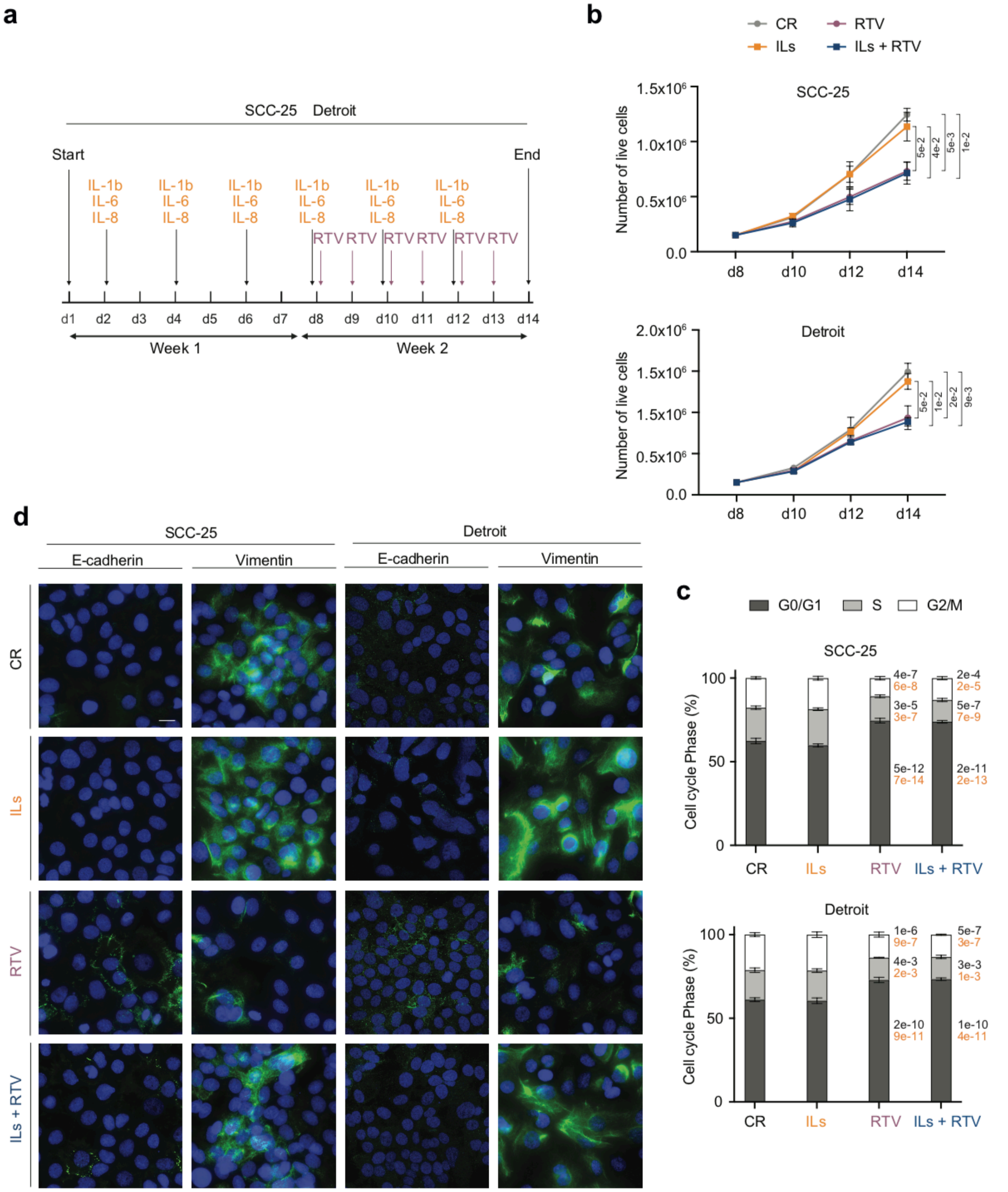

Supplementary Figure S4. Scheme of the treatments and effects of RTV on OSCCs.

(a) Schematic model of the treatment of SCC-25 or Detroit cells with combined IL-1 beta, IL-6 and IL-8 (10ng/mL each) and/or 10 μM RTV. Cells exposed to ILs dilution buffer (PBS-0.1% BSA) and/or RTV vehicle (DMSO) were employed as controls (CR). **(b)** Growth curve analysis of SCC-25 and Detroit cells treated as in **(a)** and monitored during the second week of treatment. Data presented as mean values ± SD, two-way ANOVA. **(c)** Histograms depict the percentage of SCC-25 and Detroit cells in G1, S and G2 cell cycle phases

treated as in **(a)**, on day 14 of treatment. Data presented as mean values ± SD, two-way ANOVA. Black p-values (treatment *vs* CR); yellow p-values (treatment *vs* ILs). **(d)** Representative immunofluorescence of SCC-25 and Detroit cells treated as in **(a)**, showing expression of E-cadherin (green) or Vimentin (green). Nuclei were stained with DAPI (blue). Scale Bar = 25 μm. Exact p-values are reported in the Figure.
